# Deleterious effect of short-term gavage of an ethanol extract of cogon grass (*Imperata cylindrica* L.) roots on testis and epididymal sperm quality

**DOI:** 10.14202/vetworld.2020.1311-1318

**Published:** 2020-07-13

**Authors:** Rini Widyastuti, Sigit Prastowo, Sony H. Sumarsono, Alkaustariyah Lubis, Tyagita Hartady, Mas R. A. A. Syamsunarno, Jaqueline Sudiman

**Affiliations:** 1 Laboratory of Animal Reproduction and Artificial Insemination, Department of Animal Production, Faculty of Animal Husbandry, Universitas Padjadjaran, Jl. Raya Bandung - Sumedang km. 21, West Java, Indonesia; 2 Central Laboratory, Universitas Padjadjaran, Jl. Raya Bandung - Sumedang km. 21, Jatinangor Sumedang, West Java, Indonesia; 3 Department of Animal Science, Faculty of Agriculture, Universitas Sebelas Maret, Surakarta, Indonesia; 4 Centre for Biotechnology and Biodiversity Research and Development, Universitas Sebelas Maret, Surakarta, Indonesia; 5 Physiology, Developmental Biology and Biomedical Science Research Group, School of Life Science and Technology, Bandung Institute of Technology, Bandung, West Java, Indonesia; 6 Under Graduate Medical Study Program, Faculty of Medicine, Universitas Padjajaran, Jl. Raya Bandung - Sumedang km. 21, Jatinangor Sumedang, West Java, Indonesia; 7 Department of Biomedical Science, Faculty of Medicine, Universitas Padjadjaran, Jl. Raya Bandung - Sumedang km. 21, Jatinangor Sumedang, West Java, Indonesia; 8 Department of Obstetrics and Gynecology, Faculty of Medicine, Udayana University, Jl. PB. Sudirman, Denpasar, Bali, Indonesia

**Keywords:** cogon grass root ethanol extract, epididymal sperm quality, male antifertility, reproductive hormones

## Abstract

**Background and Aim:**

Cogon grass (*Imperata cylindrica L*.) (CGG) is a herbal medicine that could be developed into a male antifertility agent. The present study aims to determine the effect of an ethanol extract of CGG roots on mice testicular activity, reproductive hormone levels, and epididymal sperm quality.

**Materials and Methods:**

This study was designed as completely randomized with three different doses, such as an ethanol extract of CGG roots at 0 (control), 90, and 115 mg/kg body weight. In total, 21 male DDY mice strain were treated with the CGG extract (by gavage) for 14 days, followed by an evaluation of reproductive organs, epididymal sperm quality, testis histology, histomorphometry, and reproductive hormone assays. All quantitative data were analyzed by analysis of variance, followed by Tukey’s *post hoc* test at α=0.05.

**Results:**

The results showed that the administration of the CGG root ethanol extract disrupted the testis interstitial area and seminiferous tubules, resulting in decreased epididymal sperm quality as well as serum testosterone levels in a dose-dependent pattern.

**Conclusion:**

Oral administration of a CGG root ethanol extract induced testicular damage, decreased epididymal sperm quality, and impaired testosterone secretion.

## Introduction

Fertility control is an exciting public health issue concerned with controlling overpopulation. Several contraceptive methods have been adopted for fertility control, but most are synthetic or costly and have many side effects. Until now, the most widely developed contraceptive methods have been for women, but side effects cannot be avoided. In contrast, the development of male contraception remains limited. In principle, male contraception acts by blocking sperm from fertilizing the egg either by physical barriers or by inhibiting spermatogenesis [1,2]. One of the known methods is vasectomy, but it has a high failure rate and lacks complete reversibility [3]. Therefore, the development of an antifertility agent that is safe, effective, reversible, and rapid-acting without affecting androgen-dependent function should be emphasized. Herbs and medicinal plants have the potential to be used as antifertility agents. Many investigations have been carried out to validate the antifertility properties of plants, such as *Bacolepis nervosa* [4], *Barleria prionitis* [5], *Actiniopteris radiata* (SW.) L., and *Selaginella bryopteris* (L.) [6]. Moreover, one study reported on 48 plants that have antifertility potential [7], but many plants have not been investigated yet. Thus, safe, acceptable, reversible, easily administrable, inexpensive, effective, and nonsteroidal antifertility candidate from a plant extract are needed.

Cogon grass (*Imperata cylindrica L*.) (CGG) is a herbal medicine that has been traditionally used to treat urinary tract infections and intestinal disorders, such as dysentery and diarrhea, myalgia, night sweats, piles, common cold [8], astringency, arthritis [9], tonic, wounds, urodynia, febrifuge [10], cancer [11], parasites, and bacteria [12], as well as to treat hypertension [13]. Based on the phytochemical screening, CGG contains alkaloids, tannins, saponins, and phytosterols [14]. *In vivo* studies have suggested that saponins and alkaloids are pro-oxidant molecules that cause oxidative damage due to the effects of free radicals (FR) and reactive oxygen species (ROS) on sperm cells and sexual glands. The expression of ROS and FR may destroy testicular germ cells through membrane damage or macromolecular degradation, resulting in sperm abnormalities [15]. Flavonoids and alkaloids induce antispermatogenic activity by reducing the diameter of seminiferous tubules and the production of spermatocytes and spermatids. Furthermore, a reduction in the numbers of immature and mature Leydig cells occur and the number of degenerating cells increases significantly. An antiprogesterone effect was also observed that was attributed to the antifertility effect [16]. The previous studies have shown that short-term gavage of a CGG extract reduces sperm concentration [17], induces abnormal sperm morphology [18], and decreases folliculogenesis in female mice [19]. Thus, CGG has the potential to be developed as an herbal antifertility agent. However, the effects of a CGG extract on testis and sperm production have not been reported.

Hence, no study has investigated the effects of a CGG root ethanol extract on testicular histomorphology and reproductive hormones, which are strongly correlated with sperm quality. Changes in testosterone level and sperm quality are standard criteria used to characterize agents that may disrupt fertility [20]. Thus, we hypothesized that a CGG root ethanol extract could be a potential male antifertility herb. Therefore, this study aimed to determine the effect of short-term gavage of a CGG root ethanol extract on epididymal sperm quality, reproductive hormone levels, and testicular histology of male mice. This is the first preliminary study to investigate the antifertility potency of a CGG root ethanol extract.

## Materials and Methods

### Ethical approval

All animal procedures in this study were approved by the Health Research Ethics Committee of the Faculty of Medicine, Universitas Padjadjaran (number 1263/UN6.C10/PN/2017).

### Cogon grass extract

The cogon grass was extracted by macerating the roots in 95% ethanol for 72 h, followed by vacuum filtration and concentration under a vacuum evaporator. The concentrated extract was suspended in 0.5% carboxymethylcellulose, and dosages of 90 and 115 mg/kg body weight (BW) were based on a previous study [18].

### Experimental animals

This study was performed at the Mouse Animal Laboratory, Faculty of Medicine and Central Laboratory, Universitas Padjadjaran. Twenty-one DDY strain mice (males; 8-12 weeks old) were obtained from PT. Biofarma (Bandung, Indonesia). The mice were housed under a 12/12 h light and dark cycle, with adequate air circulation, and had unrestricted access to water and standard feed (CV551, PT. Charoen Pokphan). The mice were assigned (in equal numbers) to three biological groups, such as a control, a group administered 0 mg/kg BW of the CGG ethanol extract (replaced with 0.5% carboxymethylcellulose) (Group A), 90 mg/kg BW (Group B), and 115 mg/kg BW (Group C) daily per oral for 14 days.

### Reproductive organ evaluation

The mice were anesthetized using isoflurane, followed by a BW measurement to evaluate the effect of the CGG root ethanol extract on reproductive organs. The mice were euthanized by cervical dislocation. The reproductive organs were isolated and removed from adherent tissues and blood, and the weights of the testis and accessory gland were recorded. The epididymis was removed to evaluate epididymal sperm quality. The ratio (index) between testis weight and BW was calculated, and the testicular tissue was stained for the histological analysis using hematoxylin and eosin (HE).

### Epididymal sperm quality evaluation

#### Sperm concentration

Epididymal sperm concentration was counted using a hemocytometer. The epididymis was finely minced with anatomical scissors in 1 mL of physiological saline in a Petri dish, and sperm was released from the epididymal tissue at room temperature for 2 min. Briefly, 1 μL of the sperm suspension was diluted in 95 μL of phosphate-buffered saline solution containing 10% formalin and 10% sucrose. Approximately 10 μL of the diluted sperm suspension was transferred to a hemocytometer and allowed to stand for 5 min. Sperm settled ware counted under a microscope at 200×, and the sperm concentration was calculated (×10^6^/mL).

#### Sperm motility

The percentage of motile sperm was determined in each sample by subjectively evaluating a drop of sperm suspension. Epididymal sperm was diluted with Tris buffer solution (3.63 g of Tris-hydroxymethyl aminomethane, 0.50 g glucose, 1.99 g of citric acid, and 100 mL of distilled water) on a prewarmed (37°C) slide, and observed under a microscope at 400×. Motility was scored from 0% to 100%.

#### Sperm viability

Sperm viability was evaluated by eosin-nigrosin staining. The sperm was mixed with eosin-nigrosin (1:1) on an object-glass, smeared, dried at room temperature, and observed under a microscope at 400×. In total, 200 sperm were evaluated; viable sperm were characterized by a pale color, while nonviable sperm was indicated by a red color on the sperm head. The proportion of viable sperm was expressed as a percentage (%) of the total number of observed sperm.

#### Sperm abnormality

Sperm morphology was observed under a microscope at 1000×. A total of 200 sperm in samples from each animal were assessed for macrocephaly, microcephaly, pinhead, no-hook, curved midpiece, bent midpiece, coiled tail, looped tail, tailless, bent tail, and curved tail, which are all abnormal sperm. We only calculated the percentage of abnormal sperm as the proportion of total abnormal sperm to total observed sperm (%).

#### Testicular histology and histomorphometric evaluation

The testis was fixed in 10% formaldehyde, embedded in paraffin, sliced to 5 μm, stained with HE and observed under a microscope. The sections were screened for histopathology and photographed. In total, 40 seminiferous tubules in each sample were examined for their diameter, width, and thickness of the epithelium. Moreover, the seminiferous tubules were scored to assess spermatogenesis using the Johnsen score, as described by Johnsen [21].

### Reproductive hormone assays

Reproductive hormones were assayed using retro-orbital-collected blood samples incubated for 15 min at room temperature. The samples were centrifuged 1500× *g* for 10 min, the serum was recovered, and the serum samples were stored at −80°C until use. The concentrations of follicle-stimulating hormone (FSH), luteinizing hormone (LH), and testosterone were determined using the mouse FSH enzyme-linked immunosorbent assay (ELISA) kit (EM1035), the mouse LH ELISA kit (EM1188), and the mouse testosterone ELISA kit (EM1850), respectively. All ELISA kits were obtained from Fine Test Company (Hubei, China), and the assays were performed according to the manufacturer’s protocol.

### Statistical analysis

This study was a completely randomized design. Data were analyzed using analysis of variance, followed by Tukey’s *post hoc* test at α=0.05. All statistical analyses were performed using the R statistical language [22].

## Results

To eliminate the effect of the CGG root ethanol extract on growth during the experiment, we evaluated the BW and reproductive organ weight of the mice in the three groups. As a result, the reproductive organ (testis, epididymis, and seminal vesicle) weights and BWs of the mice were not different after 14 days of treatment (p>0.05). However, the testis and seminal vesicle indices in both treatment groups tended to decrease compared to the control. The only difference was in the ventral prostate index, where Group C was higher (p<0.05) than the control and Group B (Table-1).

**Table-1:** Body weight and reproductive organ index of mice.

Parameter	Group		

	Control	90 mg/kg	115 mg/kg
Body weight (g)			
Initial	41.18±5.48	40.58±2.39	40.96±4.85
Final	41.58±6.10	40.36±3.59	41.43±5.46
Reproductive organ index			
Testis	0.65±0.14	0.59±0.12	0.58±0.09
Epididymis	0.27±0.07	0.24±0.03	0.26±0.05
Seminal vesicle	0.19±0.08	0.15±0.14	0.08±0.03
Ventral prostate	0.32±0.09^a^	0.31±0.14^a,b^	0.51±0.22^b^

a,b Values followed by different superscript in the same row showed significant difference (p<0.05)

**Figure-1:**
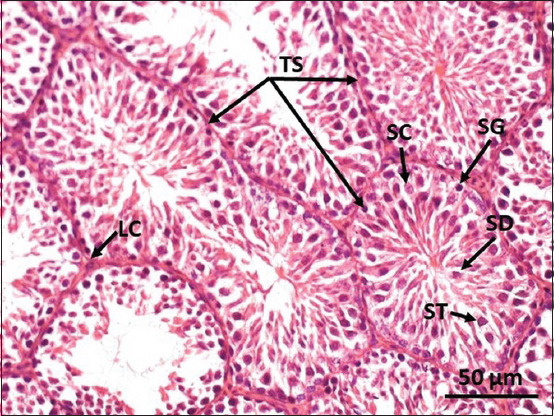
Representative photomicrograph of mouse testis tissue in the control group showing the various stages of spermatogenesis. Sertoli cells (SC) present in the seminiferous tubules (TS). Smooth muscle surrounds the seminiferous tubules in a unit. Leydig cells (LD) are spread in the interstitial space. Spermatids (SD), spermatogonia (SG), and spermatocytes (ST) are well distributed in the tubules. Leydig cells (LC) surround the seminiferous tubules.

**Figure-2:**
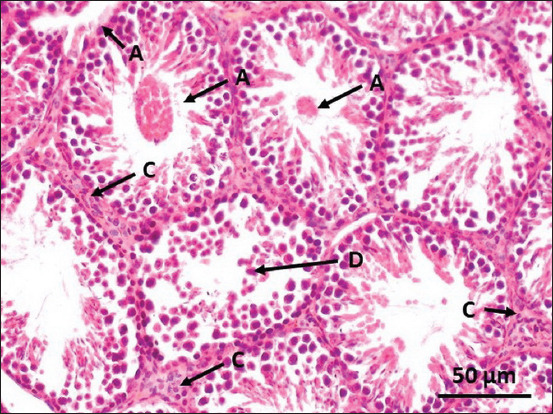
Representative photomicrograph of a mouse testis tissue from Group B (treated with 90 mg/kg body weight cogon grass [*Imperata cylindrica* L.] root ethanol extract) shows injured tubule marked by a broken epithelial wall (A). Degenerated tubules marked by the fibrin and hyaline presentation in the lumen (B). The interstitial space was filled with necrotic Leydig cells and inflammatory cells (C). Luminal diameter decreased and the germ cells arrested at the early stage of spermatogenesis (primary and secondary spermatocytes) (D).

**Figure-3:**
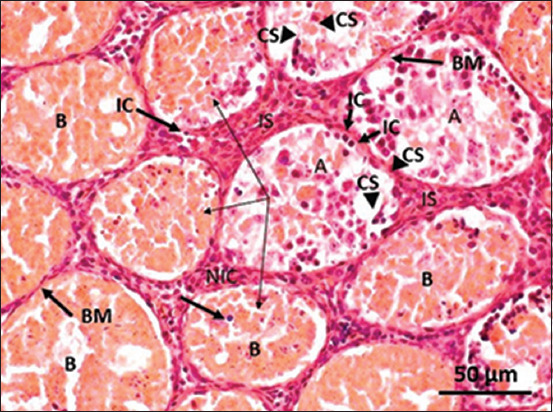
Representative photomicrograph of a rat testis tissue from Group C (treated with 115 mg/kg body weight cogon grass [*Imperata cylindrica* L.] root ethanol extract) shows severe hemorrhage of the seminiferous tubules and tubule degeneration (A); necrotic tubule (B) marked by blood infiltration filled the luminal tubule, intracellular necrosis (light arrow) and thinned basal membrane. Inflammatory cells present in the tubules and interstitial space (IC). Debris thickens in the interstitial space (IS). Some tubules are degenerating/cloudy swelling (CS) (arrowhead) and necrotic interstitial cells (NIC).

The control group assessment of testicular histology showed a compact and regular arrangement of spermatogonia in the seminiferous tubules, no lesions, and well-distributed Sertoli cells in the tubules surrounded by well-integrated smooth muscle. Leydig cells were distributed evenly in the interstitial space (Figure-1). In contrast, necrotic tubules marked by broken epithelial walls, inflammatory cells in and around the tubules, and fibrin deposits in the lumen were observed in the groups administered the CGG root ethanol extract. Necrotic Leydig cells were also observed in the interstitial space. The severity of the lesions increased with the increase in the concentration of the CGG root ethanol extract administered (Group B in Figure-2 and Group C in Figure-3).

No differences (p>0.05) were detected in testis diameter or testis area among the groups (Table-2), but a difference was found in epithelial cell height (p<0.05). The histological data generally showed a decreasing trend in the treatment groups compared to the control. The control group had the largest testis diameter and testis area compared to the treatment (B and C) groups, and this also applied to the thickness of the epithelium, in which the control was thicker than the treatment groups.

**Table-2:** Mice testis histomorphometry after the cogon grass (*Imperata cylindrica* L.) treatment.

Parameter	Group		

	Control	90 mg/kg	115 mg/kg
Tubular testis diameter (μm)	1682.42±108.21	1624.51±131.61	1500.09±103.07
Area (mm)	2253.89±0.30	2129.07±0.31	1587.41±0.31
Epithelia height (μm)	423.25^a^±88.19	419.79^a^±90.42	307.53^b^±90.13

a,b Values followed by different superscript in the same row showed significant difference (p<0.05)

Sperm concentration, motility, and viability of Groups B and C were lower (p<0.05) than the control. Moreover, we also detected a greater number of abnormal sperm in the groups that received the CGG root ethanol extract (Table-3).

**Table-3:** Mice epididymal sperm quality after the cogon grass (*Imperata cylindrica* L.) treatment.

Sperm quality	Group		

	Control	90 mg/kg	115 mg/kg
Sperm concentration (×10^6^/mL)	14.45±1.86^a^	11.12±2.71^b^	6.89±1.03^c^
Sperm motility (%)	62.29±5.07^a^	49.00±5.63^b^	26.21±5.37^c^
Sperm viability (%)	66.86±4.42^a^	58.00±3.11^b^	33.93±4.39^c^
Abnormal sperm (%)	37.29±0.81^a^	55.36±4.92^b^	64.86±2.41^c^

a,b,c Values followed by different superscript in the same row showed significant difference (p<0.05)

**Figure-4:**
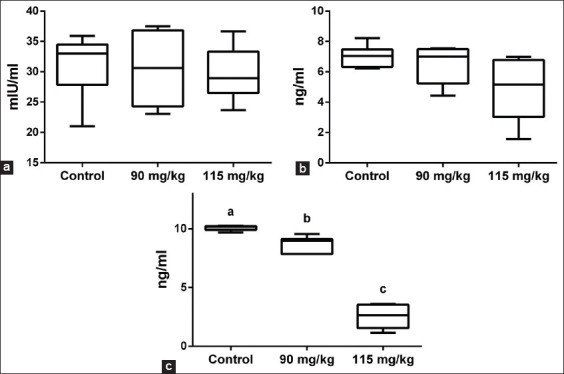
Mice reproductive hormone levels (a) FSH, (b) LH, and (c) testosterone after the cogon grass (*Imperata cylindrica* L.) treatment. Data are mean ± standard deviation, and values with different letters differ significantly (p<0.05) by group. FSH=Follicle-stimulating hormone, LH=Luteinizing hormone.

The CGG root ethanol extract significantly (p<0.05) reduced the testosterone level by 44% (Group B) and 62% (Group C) compared with the control group. FSH and LH levels were not significantly different (p>0.05) between the treatment groups and the control (Figure-4).

## Discussion

The testis is the main reproductive organ in males, and its accessory structures are responsible for maintaining sperm production. Several herbs alter testicular function, which leads to changes in sperm quantity and quality by interfering with spermatogenesis. Our results show that oral administration of the CGG root ethanol extract for 14 days to adult mice had no effect on growth between the groups (Table-1), indicating that the CGG root ethanol extract is non-toxic and was consumed safely.

The testes, epididymis, seminal vesicles, and ventral prostate are androgen-dependent organs, relying on testosterone for their function and growth [23]. Therefore, the decrease in the testis-BW ratio (index) and seminal vesicle BW ratio observed in this study may indicate decreased androgen bioavailability. Moreover, the decrease in the testis-BW ratio may result in testis atrophy, a reduction in the size of the seminiferous tubules, inhibited steroid synthesis in Leydig cells, and altered testicular structure and function [24].

The histopathological findings showed a significant change in testis structure between the treatment groups and the control group, which exerted an adverse effect on spermatogenesis. According to the histological observations, administering the CGG root ethanol extract injured the seminiferous tubules (Figures-2 and 3). A previous study showed that vacuolation of Sertoli cells is the most common response to injury [25]. Once the cells degenerate, their full function is inhibited. Germ cells will be unable to be transferred from the base to the luminal tubule. Damaged Sertoli cells are unable to synthesize essential molecules and substrates for germ cell metabolism, which impairs spermatogenesis.

Interstitial hyperplasia was enhanced and related to increasing the CGG root dose. This was described in our results (Figures-2 and 3) as enhancement of the interstitial space filled with inflammatory cells, debris, and eosinophilic granular cells. A previous study observed hyperplasia of interstitial cells (Leydig cells) marked by diffuse and consisting of an increased number of interstitial cells, which were not compressed to adjacent seminiferous tubules [26]. Diffuse hyperplasia is a physiological response to a hormone imbalance.

Meiosis and sperm production occurs in the seminiferous tubules [27]. In this study, the morphometric evaluation showed a decrease in area and a decrease in the spermatogenic cell layers (epithelial height) in a dose-dependent manner (Table-2). These decrease disrupted spermatogenesis, particularly by disturbing germinal cell mitosis, which resulted in a reduced sperm count (Table-3). Furthermore, the disturbance within the seminiferous tubules triggered the arrest of sperm maturation at different stages of development and an increase in abnormal primary sperm morphology, such as tapered heads, macro and microcephaly, amorphous heads, double heads, and knobbed acrosomal defects [28].

FSH, LH, and testosterone are sex hormones regulated by the hypothalamic-pituitary-testicular axis. FSH and LH are secreted by the anterior pituitary and bind to receptors in gonadal and non-gonadal organs. Testes contain Leydig cells that produce androgens to control FSH and LH production through inhibitory action at both the central nervous system and pituitary levels. The negative feedback effect of androgens decreases the responsiveness of the pituitary to gonadotropin-releasing hormone, thereby resulting in a decrease in LH pulse amplitude and a fall in plasma LH level. Testosterone regulates spermatogenesis by phosphorylating the cAMP response element-binding protein, and the increase in testosterone has a pivotal role in sperm quality and quantity [29]. In our study, the testosterone levels of the treated animals decreased significantly compared to the control, which was followed by a tendency for a decrease in FSH and LH levels, although it was not a significant difference (Figure-4). The decrease in testosterone may have disrupted Leydig cell function (as shown in Figures-2 and 3); however, the disruption did not induce the decrease in FSH and LH though a negative feedback mechanism. The CGG ethanol extract may act locally at the testis, and the damage may have only occurred in the tubular epithelium. The local action of CGG could be to disrupt the spermatogenesis cycle without permanently damaging the tubules [30]. Then, the epithelium would be able to regenerate during the next spermatogenic cycle within months or years. This is because the spermatogonial stem cells split infrequently and is less sensitive to toxicity compared to spermatogonia during the proliferative and developmental stages; thus, terminating the toxic compound may regenerate testis through surviving spermatogonial stem cells during seminiferous epithelial reconstruction [31].

The decrease in testosterone levels may have diminished the secretion of important substances from epididymis luminal fluid. This fluid contains ions, such as glutathione-independent prostaglandin D2 synthase, glutathione peroxidase, β-defensin, lipocalins, and cystatin-related epididymal-spermatogenic [32] that support sperm maturation and contribute directly to control cAMP concentration, which activates protein phosphorylation and sperm motility [33]. Therefore, the disturbance in the epididymis limited the energy and nutrients for sperm during maturation, resulting in diminished sperm motility (Table-3). Furthermore, the epididymal disturbance induced defects in secondary sperm morphology characterized by midpiece and tail damage or abnormal secondary sperm morphology [34]. This agrees with a previous study showing that short-term gavage of a CGG root ethanol extract induces abnormal secondary sperm morphology [18].

LH stimulates testosterone production from Leydig cells, and FSH stimulates Sertoli cells in synergy with testosterone to produce the regulatory molecules and nutrients needed to maintain spermatogenesis. FSH is correlated with Sertoli cell proliferation and testis size. In postpubertal testis, FSH, and testosterone evoke Sertoli cells signals to propagate germ cell maturation and provide antiapoptotic survival factors to regulate adhesion complexes between the germ cells and Sertoli cells. The antiapoptotic factors prevent apoptosis in spermatogonia and spermatocytes; thus, affecting sperm viability [29]. Based on our findings (Table-3), sperm viability decreased significantly in all treated animals compared to the control. The decrease in sperm viability may be related to the toxic properties of the CGG root ethanol extract. As reported in an earlier study, toxicity shrinks sperm cells and affects cell membrane permeability [35]. This disruption in membrane permeability affects sperm viability by interrupting membrane transport processes as well as the absorption of nutrients by sperm.

A phytochemical analysis of the CGG root ethanol extract revealed the presence of large chemical groups, including alkaloids, tannins, saponins, and phytosterols [14]. A previous study reported that the administration of alkaloids and triterpenoids has a negative effect on fertility [36], as they can inhibit spermatogenesis [37] and alter the morphology and histology of testes [38]. These results are comparable with a study that administered *Cissampelos capensis rhizomes*, which caused a dose-dependent increase in sperm intrinsic superoxide production leading to sperm capacitation and DNA fragmentation [39]. A similar study using withanolide-A also reported a decrease in the testosterone levels in rats, which reduced fertility and inhibited spermatogenesis [40]. Another study that administered *Catharanthus roseus* (L.) reported reduced reproductive organ weights and deceased sperm counts and serum testosterone levels in high-dose treated animals [41].

The CGG root ethanol extract had a significant effect on testosterone levels and adversely affected the semen parameters and testicular histology. These results suggest that the deleterious effects of CGG may mainly be on seminiferous tubules without affecting hypothalamic-pituitary function. Further investigations are needed to test the effect of the CGG root ethanol extract on mating rate and the reversibility of testicular damage and sperm quality after long-term administration to justify that it is a good candidate as a male antifertility agent. In addition, further research about the appropriate dose and potential for toxicity is required to fully evaluate the effects of the CGG root ethanol extract.

## Conclusion

This study revealed that administering an ethanol extract of CGG roots to male mice induced histoarchitectural and testicular disturbances that impaired testosterone levels and sperm quality. The CGG ethanol extract is reported to be non-toxic and could be a safe drug choice. Further studies, based on the current results, could lead to a new herbal candidate with antifertility properties.

## Authors’ Contributions

RW, SP, SHS, and JS contributed to the design and carried out the experiment, data analysis, and interpretation. Sample and histopathological analyses were performed by AL, TH, and MRAAS. RW and SP wrote the manuscript in consultation with all of the authors. All authors have revised and approved the final manuscript.
